# Supplementary material for: Characterization of Endoplasmic Reticulum (ER) in Human Pluripotent Stem Cells Revealed Increased Susceptibility to Cell Death upon ER Stress
Source: Cells. 2020 Apr 26;9(5):1078. doi: 10.3390/cells9051078 (PMC7291192; doi:10.3390/cells9051078)
Supplement: Supplementary file 1 [file cells-09-01078-s001.zip › Figure_S1.docx]

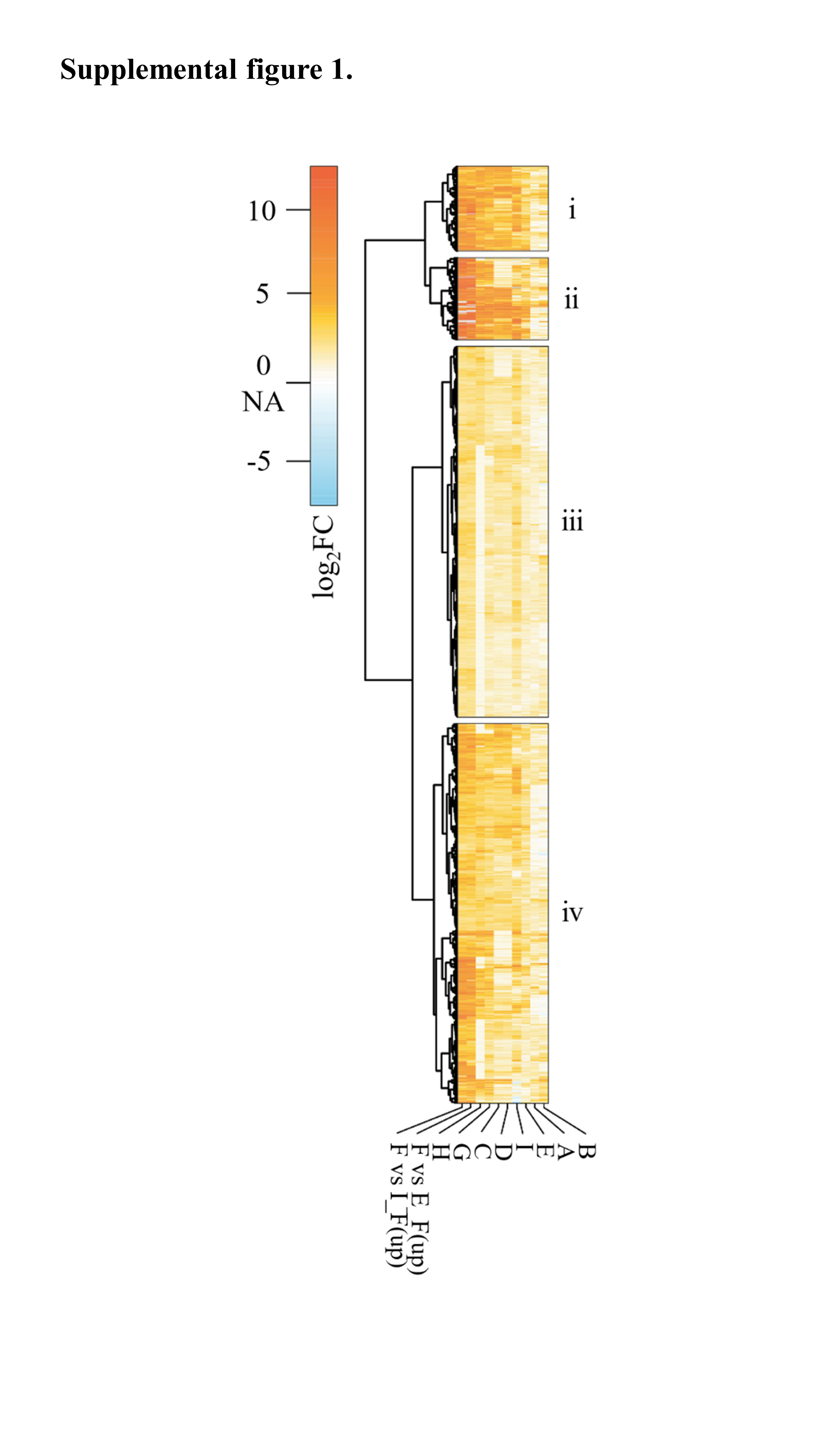


**Figure S1.** Heat-map showing the log_2_ fold-change value for the four generated clusters. GEO data used are abbreviated as follows; A: GSE54186, ESC (H1) vs. differentiated Fibroblasts; B: GSE54186, ESC (H9) vs. differentiated Fibroblasts; C: GSE33298, ESC vs. Fibroblasts; D: GSE33298, IPSC vs. Fibroblasts; E: IPSC vs. Fibroblast; G: GSE24487, IPSC vs. Fibroblast; H: GSE24487, ESC vs. Fibroblast; I: GSE20750, IPSC vs. Fibroblast. All four clusters are significantly down-regulated in the hPSC group. Although the fold change distribution of cluster iii is moderate compared to the dynamic differences in other clusters, these data clearly suggest that ER and Golgi are not as functional in hPSCs.
