# Supplementary material for: Characterization of Endoplasmic Reticulum (ER) in Human Pluripotent Stem Cells Revealed Increased Susceptibility to Cell Death upon ER Stress
Source: Cells. 2020 Apr 26;9(5):1078. doi: 10.3390/cells9051078 (PMC7291192; doi:10.3390/cells9051078)

**Figure S2.** Western blot showing the expression level of the UPR sensors protein IRE1-alpha and ATF6. Expression levels of IRE1-alpha, ATF6 and actin in ESCs, iPSCs and fibroblasts were assessed by western blotting. Each wild type cells lysates are used as samples.


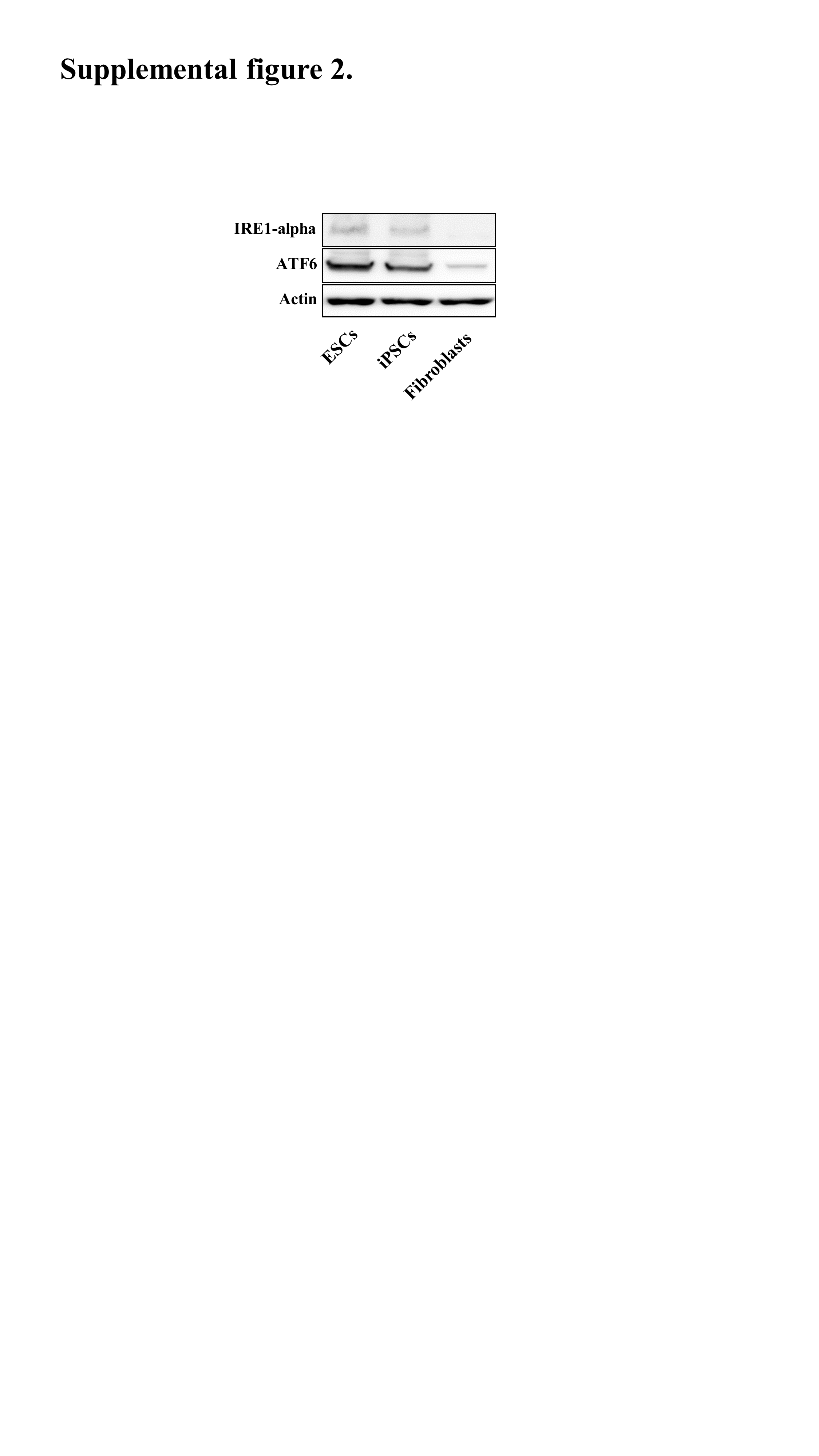

Supplement: Supplementary file 1 [file cells-09-01078-s001.zip › Figure_S2.docx]
